# The Adult Livers of Immunodeficient Mice Support Human Hematopoiesis: Evidence for a Hepatic Mast Cell Population that Develops Early in Human Ontogeny

**DOI:** 10.1371/journal.pone.0097312

**Published:** 2014-05-12

**Authors:** Marcus O. Muench, Ashley I. Beyer, Marina E. Fomin, Rahul Thakker, Usha S. Mulvaney, Masato Nakamura, Hiroshi Suemizu, Alicia Bárcena

**Affiliations:** 1 Blood Systems Research Institute, San Francisco, California, United States of America; 2 Laboratory Medicine, University of California San Francisco, San Francisco, California, United States of America; 3 Liver Center, University of California San Francisco, San Francisco, California, United States of America; 4 Biomedical Research Department, Central Institute for Experimental Animals, Kawasaki, Japan; 5 Department of Obstetrics, Gynecology and Reproductive Sciences, Institute for Regeneration Medicine, University of California San Francisco, San Francisco, California, United States of America; European Institute of Oncology, Italy

## Abstract

The liver plays a vital role in hematopoiesis during mammalian prenatal development but its hematopoietic output declines during the perinatal period. Nonetheless, hepatic hematopoiesis is believed to persist into adulthood. We sought to model human adult-liver hematopoiesis by transplantation of fetal and neonatal hematopoietic stem cells (HSCs) into adult immunodeficient mice. Livers were found to be engrafted with human cells consisting primarily of monocytes and B-cells with lesser contributions by erythrocytes, T-cells, NK-cells and mast-cells. A resident population of CD117^++^CD203c^+^ mast cells was also documented in human midgestation liver, indicating that these cells comprise part of the liver's resident immune cell repertoire throughout human ontogeny. The murine liver was shown to support human multilineage hematopoiesis up to 321 days after transplant. Evidence of murine hepatic hematopoiesis was also found in common mouse strains as old as 2 years. Human HSC engraftment of the murine liver was demonstrated by detection of high proliferative-potential colony-forming cells in clonal cultures, observation of CD38^−^CD34^++^ and CD133^+^CD34^++^ cells by flow cytometry, and hematopoietic reconstitution of secondary transplant recipients of chimeric liver cells. Additionally, chimeric mice with both hematopoietic and endothelial reconstitution were generated by intrasplenic injection of immunodeficient mice with liver specific expression of the urokinase-type plasminogen activator (uPA) transgene. In conclusion, the murine liver is shown to be a hematopoietic organ throughout adult life that can also support human hematopoiesis in severely immunodeficient strains. Further humanization of the murine liver can be achieved in mice harboring an uPA transgene, which support engraftment of non-hematopoietic cells types. Thus, offering a model system to study the interaction of diverse human liver cell types that regulate hematopoiesis and immune function in the liver.

## Introduction

The liver is the primary site of hematopoiesis during the latter half of human embryonic development through midgestation [1], [2]. Fetal liver hematopoiesis is highly skewed towards erythropoiesis, being comprised of a plethora of erythroid progenitors and immature red cells [3], [4]. Multilineage hematopoiesis does occur in the liver as evidenced by the presence of myeloid and lymphoid progenitors in addition to the hematopoietic stem cells (HSCs) found in the developing liver [5]–[7]. At the start of the second trimester of gestation hematopoiesis also begins in the bone marrow (BM), which eventually surpasses the liver as the primary site of hematopoiesis in the second half of gestation [8], [9]. Although liver hematopoiesis wanes early in human ontogeny, remnants of hematopoiesis are believed to persist into adulthood.

In young-adult mice (6–8 weeks old) the presence of a resident population of hematopoietic cells has been demonstrated in the liver with the characteristics of HSCs and early progenitors [10]. These cells had hematopoietic colony-forming potential in vitro and could form splenic colonies when transplanted into lethally-irradiated recipients. The adult murine liver was also shown to be a site of extrathymic T- and NK-lymphopoiesis arising from a population of parenchymal CD117^+^ (c-kit) cells [11], [12]. Moreover, transplant experiments demonstrated long-term multilineage hematopoietic reconstitution by purified CD117^+^ or lineage^−^ SCA-1^+^ CD117^+^ liver-derived cells indicating the presence of a population of HSCs [11], [13]. In addition, a highly enriched population of HSCs, defined by low staining with the dye Hoechst 33342, has also been described in the liver [14]. These cells were similar to those found in the BM but, interestingly, do not express CD117, in contrast to the earlier reports. This liver cell population could, nonetheless, arise from transplanted BM cells.

Human hematopoietic progenitors have been isolated from adult liver biopsies and resections based on their expression of CD34 [15]. About half of these CD34^+^ liver cells expressed the common leukocyte antigen CD45 indicating that they are hematopoietic in nature, as opposed to being endothelial cells or some other non-hematopoietic CD34^+^ cell type. CD34^+^ liver cells were also found to express CD38 and HLA-DR, both antigens found on adult hematopoietic progenitors, but not stem cells [16]. Myeloid, erythroid and mixed lineage colony-forming cells (CFCs) were detected in cultures further indicating the presence of hematopoietic progenitors [15]. Moreover, the presence of HSCs in the human adult liver is strongly suggested by the presence of cells with the phenotypic profile of HSCs, CD38^−^CD90^+^CD34^+^ and HLA-DR^low^CD34^+^, capable of hematopoietic engraftment of immunodeficent mice [17].

Further evidence that HSCs reside in adult liver derives from observations of blood chimerism after liver transplantation. BM biopsy after orthotopic liver transplantation revealed engraftment by CD34^+^CD38^−^HLA-DR^low^ cells, possibly representing HSCs, as well as lineage-committed progenitors of donor origin [18]. As HSCs are normally found in small numbers in the peripheral circulation, blood trapped within the liver at the time of transplant was a possible source of the engrafted HSCs. However, perfusion of the liver prior to transplant likely depleted the number of blood-borne HSCs. Other transplant cases have also resulted in hematopoietic chimerism and support the conclusion that the adult liver harbors HSCs [19], [20].

Questions remain surrounding the role of hematopoiesis in the adult liver. Do HSCs reside in the liver throughout ontogeny and to what degree to they contribute to hematopoiesis and the in situ development of blood cells found in the liver, thereby contributing to the overall immunological functions of the liver [10]? Immunodeficient NOD.Cg-Prkdc^scid^ Il2rg^tm1Wjl^/SzJ (NSG) mice humanized by transplantation of HSCs offers a small animal model to study human hematopoiesis. In one previous study only 0.18% human cells were detected in the livers of NOD.Cg-Prkdc^scid^ (NOD-SCID) mice transplanted with CD34^+^ umbilical cord blood (UCB) cells [21]. Similar transplants performed in Rag2^−/−^γ_c_^−/−^ mice gave rise to the presence of human dendritic cell populations in the liver [22]. More recently, we observed by flow cytometry the presence of human hematopoietic cells, including candidate HSCs, in the livers of adult transplanted NSG mice [23]. Herein, we report on an extensive evaluation of human hematopoietic cell populations that can be found in the livers of chimeric mice. We demonstrate that the liver harbors a diversity of mature blood cells representing lymphoid, erythroid and myeloid cell types. One notable finding was the presence of a population of human mast cells in the murine livers. The existence of mast cells in fetal hematopoietic tissues used as grafts was also investigated. Chimeric mice were evaluated for the presence of adult liver hematopoiesis and evidence that the adult murine liver can support a population of human HSCs.

## Materials and Methods

### Ethics Statement

Human fetal tissues were obtained from elective abortions and UCB was obtained from live births with the written consent of the women undergoing the procedures at San Francisco General Hospital and Moffitt Hospital, University of California San Francisco, USA. This research was performed with the approval of the University of California San Francisco's Committee on Human Research. All specimens were anonymous.

Animal research was performed with approval of the Institutional Animal Care and Use Committee at ISIS Services LLC (San Carlos, CA, USA), protocol numbers IAC 1101/ANS 1515, IAC 1438/ANS 1795, IAC 1294/ANS 1665, IAC 1179/ANS 1578 and IAC 1567/ANS 1908. Additionally, some experiments were performed at the University of California San Francisco with approval of the Committee for Animal Research at that institute, protocol number AN079387. Every effort was made to reduce the number of animals used for this study through tissue-sharing and coordinated analysis of transplanted mice to simultaneously investigate multiple experimental parameters. All animals were adults (≥ 8 weeks of age) at the time of sacrifice; the weights of the animals varied depending on age and sex, and likely fell in a range of 20–40 g.

For intra-splenic transplants, mice were deeply anesthetized by inhaled vaporized-isoflurane. Mice received humane care according to the criteria outlined by the National Research Council's Institute of Laboratory Animal Resources in the "Guide for the Care and Use of Laboratory Animals". A completed ARRIVE (Animal Research: Reporting of *In Vivo* Experiments) checklist provided by the National Centre for the Replacement, Refinement and Reduction of Animals in Research is found in supplementary data file Checklist S1.

### Human tissues and cell isolation

The age of each fetus was estimated based on foot length and ranged between 15 and 24 weeks' gestation. Human fetal bone marrow (hFBM), light-density fetal liver (LDFL) and light-density UCB depleted of mature blood cells; i.e. lineage (Lin) antigens: CD3, CD14, CD19, CD20, CD56 and CD235a; were used as sources of hematopoietic cells [24]. Light-density, erythrocyte-depleted or Lin^−^ cells were prepared from human fetal livers (hFL) as previously described [25] for flow cytometric analysis. Hematopoietic precursors, intended for transplantation, were sorted from Lin^−^ LDFL stained with CD34-allophycocyanin (APC) and CD45-fluorescein isothiocyanate (FITC) monoclonal antibodies (mAbs) for isolation of CD34^++^CD45^+^ cells. Alternatively, these cells were stained with CD34-APC and CD38-phycoerythrin (PE) for isolation of CD38^−^CD34^++^ cells that are enriched in HSCs [25]. Cell sorting was performed using a BD FACSAria III (BD Biosciences, San Jose, CA, USA). All mAbs used in this study are listed in Table S1.

### Generation of human-mouse hematopoietic chimeras

This study was initiated using male and female NOD-SCID mice. In early experiments, NOD-SCID mice received 300 cGy γ-irradiation and donor cells were injected together with 2×10^7^ lethally-irradiated (3000 cGy γ-irradiation) hFBM cells used as carrier cells.

NSG mice were used instead of NOD-SCID mice once the NSG strain became available to us. Indeed, the possibility of achieving higher levels of human chimerism in NSG mice led to most experiments being performed using this strain of mice. Both male and female NOD-SCID and NSG mice were used as indicated in the Results section and figure legends. Breeder pairs of both strains were purchased from Jackson Laboratories (Bar Harbor, ME) and raised at our institute. These mice were housed under specific-pathogen free conditions and given antibiotic-laced feed after irradiation and/or human cell transplantation as previously detailed [23]. Hematopoietic chimeras were created by transplantation of human hematopoietic precursors into X-ray irradiated (175–275 cGy) NSG mice by intravenous (i.v.) tail vein injection.

Additionally, male and female NOD.Cg-Prkdc^scid^ Il2rg^tm1Sug^ Tg(Alb-Plau)11-4/ ShiJic (uPA-NOG) mice [26] were used as recipients of intra-splenic transplants delivered without prior irradiation or carrier cells.

Any mice that fell ill as a result of natural causes or due to experimental procedures were sacrificed according to protocol approved guidelines. At the time of sacrifice, animals were excluded from the study if splenic, hepatic or thymic tumor formation was observed or if they exhibited signs of graft versus host disease (GvHD) such as severe hair-loss or gross splenomegaly.

### Tissue harvest and cell preparation

Livers and spleens were harvested from mice after CO_2_ asphyxiation and cervical dislocation. BM was obtained by flushing both femora. In some experiments peripheral blood was harvested by orbital enucleation of mice given inhalation anesthesia and collection of the blood in heparinized tubes. Hematopoietic tissues were then collected from these mice after cervical dislocation. Hematopoietic cells were isolated from livers and spleens by passing these organs through 100 µ cell strainers (BD Biosciences) and isolating light-density (≤1.077 g/ml) cells as previously described [23].

For experiments on old-age untransplanted mice, light-density BM cells were prepared for comparison to similarly prepared liver samples. Balb/cJ, C57BL/6J and C3H/HeJ mice were bought from Jackson Laboratories as young adults and housed at our institute.

Livers harvested from uPA-NOG mice transplanted by intra-splenic transplantation were disrupted by enzymatic digestion, as described [27], to preserve the viability of non-hematopoietic liver cells. Digested liver cell suspensions were held in 50ml tubes and allowed to separate, for approximately 5 minutes, into quickly-settling high-density and the remaining low-density cells. These two cell fractions were analyzed separately.

Liver cell counts were performed using a hemocytometer with trypan blue (Life Technologies, Grand Island, NY) staining for exclusion of dead cells or with a Scepter Handheld Automated Cell Counter with 60 µm sensors (EMD Millipore Corporation, Billerica, MA, USA).

### Phenotypic analysis

Washed cells were suspended in blocking buffer consisting of PBS supplemented with 0.01% NaN_3_ (Sigma Chemical Co., St. Louis, MO, USA) and 5% normal mouse serum. When mouse cells were present, 2 µg/mL rat anti-mouse CD16/CD32 mAb (BioLegend, San Diego, CA) was also added to the blocking buffer to reduce non-specific binding of mAbs. Cells were stained with fluorochrome-labeled mAbs (Table S1) for 30 minutes. Samples were washed twice with PBS supplemented with 0.3% bovine serum albumin (Roche Diagnostic Corporation, Indianapolis, IN, USA) and 0.01% NaN_3_ then suspended in the same solution containing 2 µg/mL propidium iodide (PI; Invitrogen, Carlsbad, CA, USA) used to stain dead cells. Sample analysis was performed using an LSR II flow cytometer (BD Biosciences).

Data were analyzed using FlowJo software, version 9 (Tree Star, Inc., Ashland, OR, USA). Only single live cells were considered for analysis based on gating PI^−^ events and doublet discrimination (Fig. S1A). Additionally, human cells were identified among murine cells by their expression of CD59 and a lack of expression of the murine antigens TER-119, CD45 and H-2K^d^ [23]. All flow cytometric data on human cells harvested from murine tissues represent events gated in this manner.

For the analysis of human mast cells, an additional light-scatter gate was used to focus in on events with a low side-light scatter and moderate forward scatter that enriched for CD117^+^CD203c^+^ mast cells. The percentages of positive events in univariate comparisons made on mast cells, as well as other cell populations, were calculated using super-enhanced Dmax subtraction using isotype-matched antibodies for negative controls. Results from these calculations are presented on over-layered histogram plots showing antigen and control staining.

Murine hematopoietic precursors were analyzed from light-density BM and liver cells using the same live single-cell gating strategy as described above. Additionally, cells expressing mature murine Lin markers (CD3, CD11b, CD45R, Gr-1 and TER-119) were excluded from analysis.

### Hematopoietic colony-forming cell (CFC) assay

Human myeloid precursors, low proliferative potential (LPP)-CFCs and high proliferative-potential (HPP)-CFCs, were assayed as previously described [28] with some minor modifications. No serum was added to the cultures, instead a serum-deprived medium was used as detailed elsewhere [2]. Additionally the recombinant human cytokines, which do not support the growth of murine progenitors, were used at the following concentrations: kit ligand (KL) was used at 50 ng/ml (R&D Systems, Minneapolis, MN, USA), granulocyte-macrophage colony-stimulating factor (GM-CSF) was used at 20 ng/ml (Immunex Corporation, Seattle, WA, USA) and interleukin-3 (IL-3) was also used at 20 ng/ml (Amgen, Inc., Thousand Oaks, CA, USA).

Murine colony-forming units-culture (CFU-c) were assayed in triplicate 1 ml cultures as previously reported [29] with minor modification: serum-deprived medium [2] was used as the base medium with 20% fetal bovine serum (FBS; Stemcell Technologies, Canada) added. Growth was supported by the following recombinant cytokines rat kit ligand (rrKL; Amgen), mouse interleukin-3 (rmIL-3; R&D Systems) and mouse granulocyte-macrophage colony-stimulating factor (rmGM-CSF; BioLegend, San Diego, CA). Following 7 days incubation, colonies are enumerated and total CFU-c were calculated for each liver based on cell recoveries and the frequency of CFU-c detected.

### Immunofluorescence staining and epifluorescence microscopy

Pieces of mouse liver were fixed, embedded, sectioned with a cryostat and stained as previously described [30]. Primary and secondary antibodies used to stain the sections are listed in Table S1. Slides were covered with ProLong Gold antifade reagent with 40′,6-diamidino-2-phenylindole (DAPI) (Life Technologies, Grand Island, NY, USA). Images were analyzed with a Leica CTR6500 (Leica Microsystems, Buffalo Grove, IL, USA). Colocalization analysis was performed using iVision software (BioVision Technologies, Exton, PA, USA).

### Statistical analyses

Data charting and statistical analysis was performed using Aabel 3 software (Gigawiz Ltd. Co. OK, USA). The numbers of animals evaluated are detailed in results section and figure legends. A bar chart is used to display mean ± standard error of CFC numbers. Cell numbers recovered from transplanted mice are presented on box plots with medians indicated by notches and whiskers extending to extreme data points. The 2-tailed Mann-Whitney U-test was used to determine the significance of differences between groups of transplanted mice. A 2-tailed unpaired t-test was used to compare measurements of murine cells from untransplanted mice. A P-value of ≤0.05 is considered significant.

## Results

### Mature human blood cells are found in the murine liver

**Figure 1:**
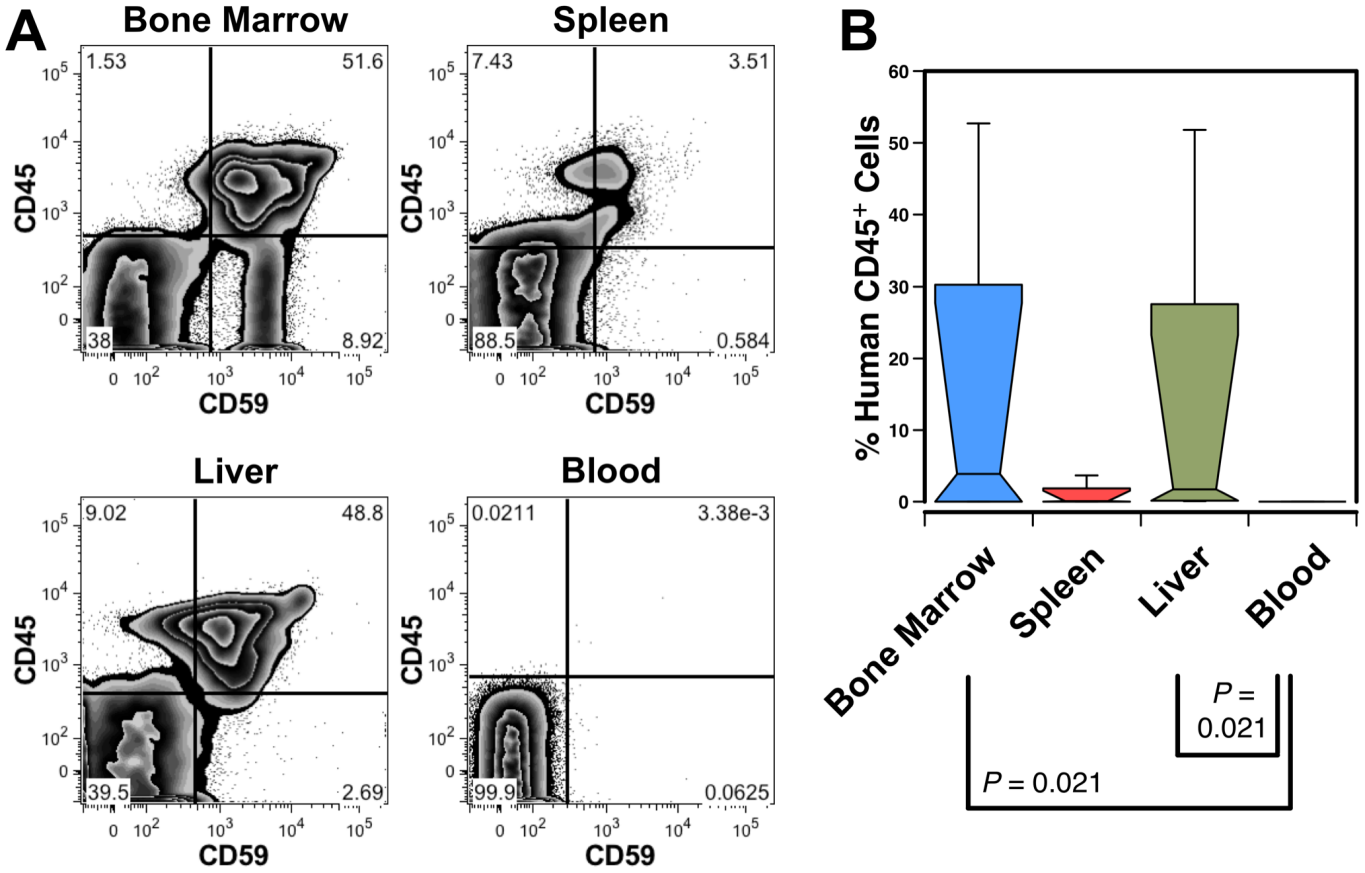
Engraftment of the murine liver with human blood cells. (A) A NOD-SCID mouse transplanted with human CD34^++^CD45^+^ hFL cells was analyzed 71 days after transplant revealing high level engraftment of human CD45^+^CD59^+^ cells in the BM, spleen and liver; no clear indication of circulating human cells was seen in the blood. (B) Box plot showing the median and range of human leukocyte engraftment in 4 transplanted NOD-SCID mice from 2 independent experiments analyzed at 67 and 71 days post transplant. Significant differences among all possible pair-wise comparisons are indicated.

To determine if the murine liver can support human hematopoiesis, we first sought to confirm that human cells found in the liver are not simply the result of trapped peripheral-blood cells. Engraftment was evaluated in NOD-SCID mice transplanted with CD34^++^CD45^+^ hFL cells. Analysis of the tissue distribution of human cells in a mouse with a high level (>50%) of BM engraftment shows liver engraftment but almost no human cells in peripheral blood (Fig. 1A). Similar results were observed in 3 additional mice (Fig. 1B), as well as in an experiment performed using NSG mice (data not shown). This indicates that the bulk of the human cells harvested from the liver must reside in this organ, as opposed to being free-floating blood cells.

**Figure 2:**
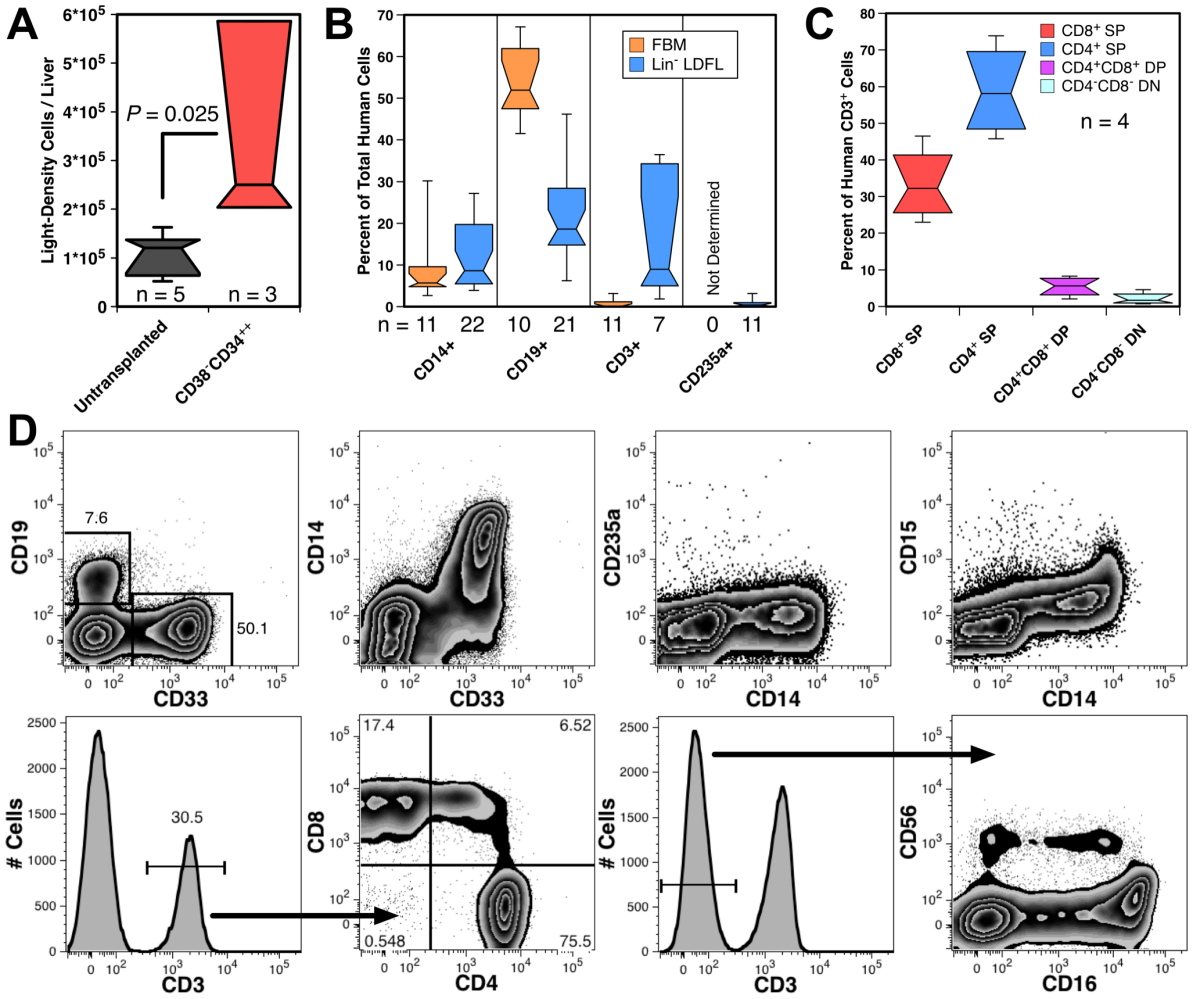
Mature human blood cells in the murine liver. (A) Significantly more light-density liver cells were recovered from 3 mice transplanted with CD34^++^CD38^−^ hFL cells than from untransplanted NSG mice. (B) Myeloid, lymphoid and erythroid engraftment observed 68-166 days after transplantation with hFBM or Lin^−^ LDFL cells. (C) Distribution of T-cell subsets among CD3^+^ T-cells in mice transplanted with hFBM cells. The numbers (n) of animals evaluated are indicate in the 3 box plots. (D) Flow cytometric analysis of light-density liver cells pooled from 3 NSG mice transplanted with CD34^++^CD38^−^ hFL cells were analyzed 144 days after transplantation showing multilineage hematopoietic engraftment. T-cell subsets were evaluated by gating on CD3^+^ cells as indicated. CD56^+^ NK cells were defined by a low side-light scatter gate (not shown) and their lack of CD3 expression. Numbers shown in the graphs represent the percentages of gated events among all CD59^+^ human cells.

The lineage composition of the human blood cells found in the liver was analyzed from transplanted NSG mice. The number of light-density liver cells recovered from transplanted mice was higher than that of untransplanted mice (Fig. 2A). Mice transplanted with 2 different preparations of hFBM and 5 preparations of Lin^−^ LDFL all had human CD14^+^ monocytes and CD19^+^ B-cells present in their livers (Fig. 2B). CD3^+^ T-cells were also detected in 15 of 18 animals, the majority of which were CD4^+^ or CD8^+^ single-positive (SP) T-cells, but low frequencies of double positive (DP) and double negative (DN) T-cells were also observed (Fig. 2C). As human T-cell engraftment has been associated with GvHD in immunodeficient mice, we avoided inclusion of data from animals with indications of GvHD. Gross signs of GvHD (severe hair-loss and splenomegaly) were rarely observed and only long after transplant. Fig. S1 shows an example of hematopoietic engraftment of the liver, associated with GvHD, observed 321 days after transplant.

To ascertain if the mature blood cells developed from transplanted HSCs and not from long-lived mature cells present in the graft, we isolated highly enriched HSCs, CD38^−^CD34^++^ cells, and analyzed engraftment 144 days after transplant. Liver cells pooled from transplanted mice contained a mixture of myeloid and lymphoid cells (Fig. 2D). These included CD33^+^ myeloid cells, most of which were CD14^+^ monocytic cells. Only a small number of CD15^+^ granulocytes and CD235a^+^ erythrocytes, which were likely depleted by the density separation procedure, were detected. Lymphoid cells were predominantly CD19^+^ B-cells, but SP, DP and very few DN CD3^+^ T-cells were also observed. CD56^+^CD3^−^ NK-cells were detected, the majority of which expressed CD161 (not shown) and included both CD16^+^ and CD16^−^ subsets (Fig. 2D).

### The liver contains a resident population of mast cells

**Figure 3:**
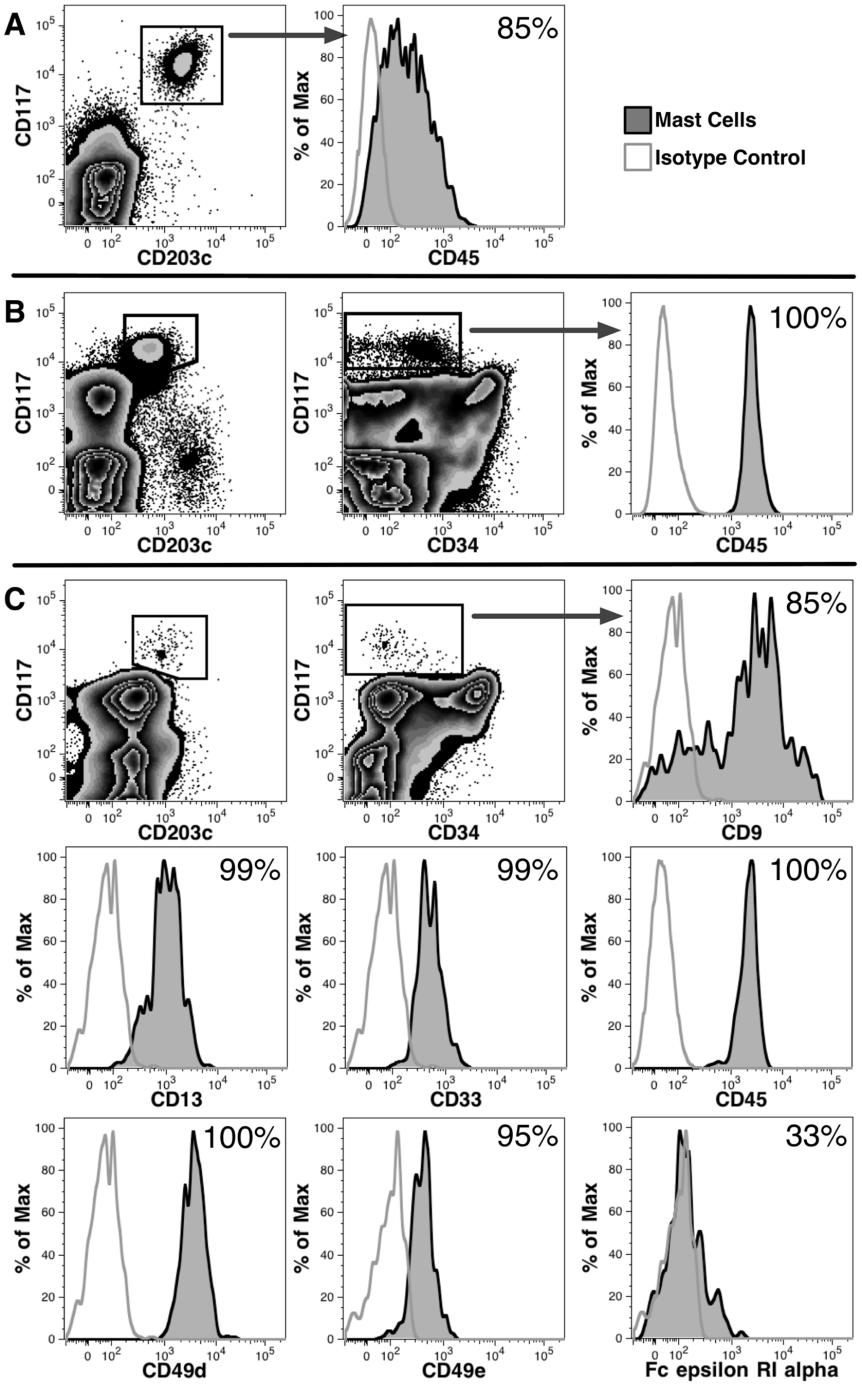
Mast cells are present in the liver of chimeric mice and in human prenatal development. (A) Light-density liver cells, pooled from 9 chimeric mice, were analyzed 89 days after transplantation with hFBM cells. CD117^++^CD203c^+^ mast cells are indicated by the rectangular gate, which also express low levels of CD45. (B) CD117^++^CD203c^+^ mast cells were found in human hFBM at 20 weeks' gestation, which also express low levels of CD34 and CD45. (C) CD117^++^CD203c^+^ mast cells were observed in hFL at 18 weeks' gestation. Antigen expression on CD117^++^ mast cells, gated as indicated in the dot plot, are shown using histograms. The frequency of positive cells, relative to isotype controls shown in grey outline, are indicated in each plot.

Mast cell engraftment of the liver has not been studied in humanized mice. Since the adult liver contains a resident population of mast cells [31], we investigated if these cells could develop in chimeric NSG mice. CD117^++^CD203c^+^ cells, expressing low levels of CD45, were found among liver cells harvested 3 months after transplantation with hFBM cells (Fig. 3A).

Unlike other granulocytic lineages, mature mast cells are capable of proliferation. Thus, it was unknown if the mast cells detected in chimeric mice represented mast cells present in the graft and/or if they were the progeny of hematopoietic precursors. Therefore, we first studied if mast cells are present in midgestation hematopoietic tissues. Indeed, CD117^++^CD203c^+^ mast cells were observed among hFBM (Fig. 3B) and hFL cells (Fig. 3C). These cells mostly lacked expression of CD34, although a few CD34^+^CD117^++^ cells were detectable – possibly representing a committed progenitor population. Phenotypic analysis of mast cells from hFL (Fig. 3C) revealed that these cells expressed CD9, CD13, CD33, CD45, CD49d, CD49e and low levels of FcεRIα. Lymphoid antigens such as CD3, CD19 and CD56 were not expressed, and negligible or low levels of CD15 and HLA-DR were observed (data not shown). This antigen profile is consistent with CD117^++^CD203c^+^ representing a population of mast cells that is present in both the human fetal hematopoietic tissues and in the livers of transplanted mice [32]–[34].

To determine if NSG mice support the development of human mast cells, we transplanted mice with 2×10^2^ CD38^−^CD34^++^ cells. CD117^++^CD203c^+^ mast cells were detected in these mice 187 days after transplantation, demonstrating mast cell development in NSG mice from an enriched HSC population (data not shown).

### Human hematopoiesis in the murine liver

**Figure 4:**
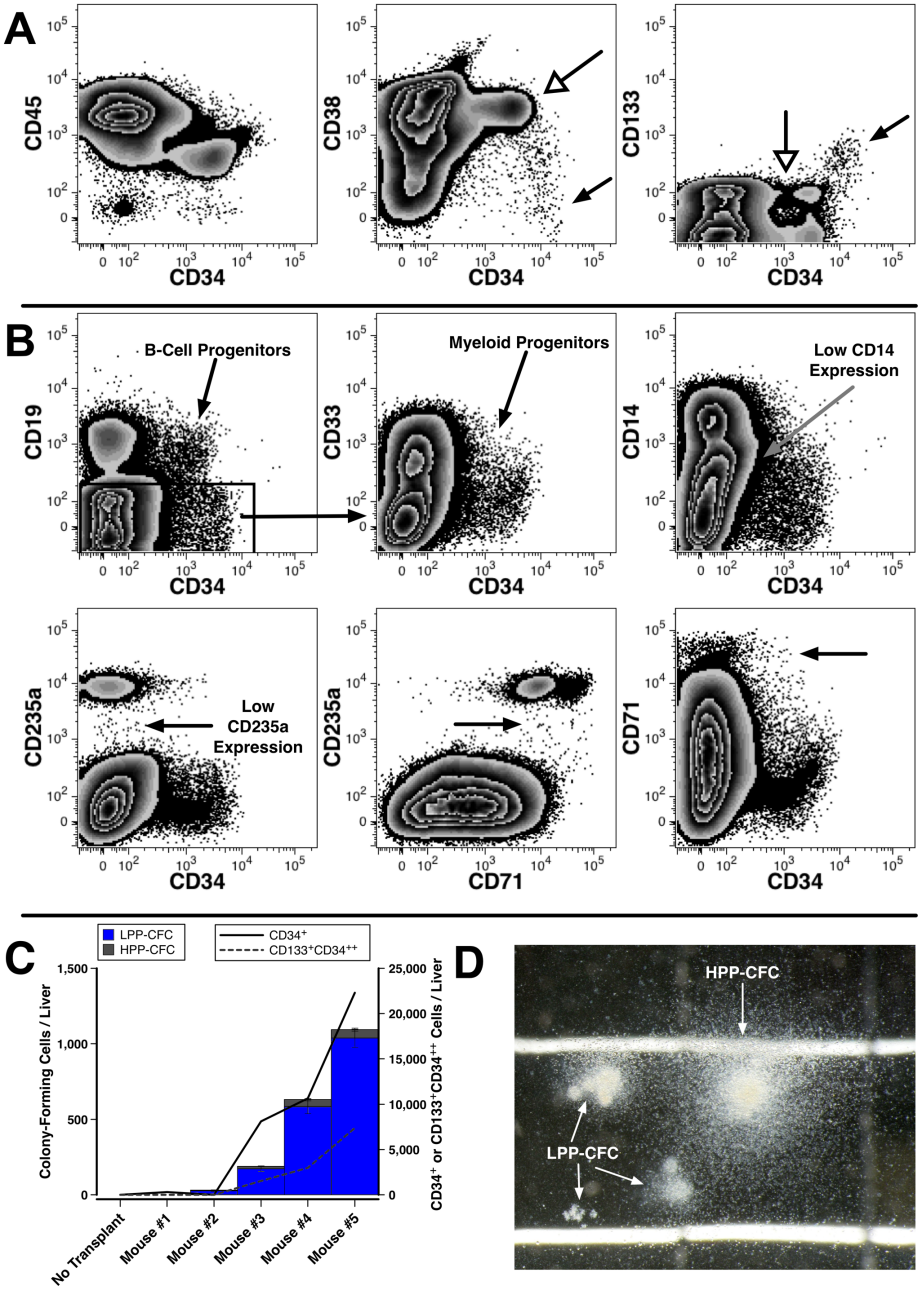
Human hematopoiesis in the livers of mice transplanted with fetal cells. (A) Hematopoietic precursors are present in the liver of a mouse analyzed 130 days after being transplanted with 1×10^6^ hFL cells. Filled arrows identify CD38^−^CD34^++^ and CD133^+^CD34^++^ cells, possible HSCs, whereas open arrows point to committed progenitors. (B) Various committed hematopoietic progenitor populations are evident in the liver including B-cell progenitors and myeloid progenitors shown from among CD19^−^ human cells.The bottom row of data shows immature erythroid cells that express low levels of CD235a and high levels of CD71 as indicated by the arrows. Data are from 4 pooled livers analyzed 148 days after transplantation with 2×10^5^ Lin^−^ hFL cells. (C) HPP-CFC and LPP-CFC responsive to human-specific cytokines were assayed from the light-density livers cells harvested from 5 transplanted and 1 untransplanted NOD-SCID mice. Mice were analyzed 30 days after transplant with 1×10^7^ hFBM cells of 23 weeks' gestation. Lines indicate the total number of CD34^+/++^ and CD133^+^CD34^++^ cells (right axis) shown on top of a bar chart of colony numbers (left axis). (D) A photomicrograph of representative myeloid colonies grown from liver cells shows both a large HPP-CFC-derived colony and smaller LPP-CFC-derived colonies. The size of the colonies can be gauged from the 2 mm grid shown in the background.

We next sought to determine if human hematopoiesis occurs in the livers of transplanted NSG mice by examining markers that define stages of hematopoietic differentiation. CD34^+^CD45^+^ cells were observed in the liver (Fig 4A) and putative HSCs expressing high levels of CD34, low levels of CD133 and lacking CD38 expression were also observed. Committed progenitors were observed based on the presence of CD38^+^ and CD133^−^ cells expressing a spectrum of CD34.

Lineage-committed progenitors were observed including CD19^+^CD34^+^ B-cell progenitors and myeloerythroid progenitors expressing CD33 but not CD19 (Fig 4B). We also observed a spectrum of CD14 expression, suggestive of monocytic cells at various stages of differentiation. We were able to document the presence of erythroid precursors in livers coming from mice with high levels of hematopoietic chimerism (mean  =  61% human cells in the BM, n  =  4). Immature CD235a^low^CD71^++^ erythroid cells were seen as well as some CD34^+^ cells expressing high levels of CD71.

### Hematopoietic colony formation by liver cells

Human myeloid CFC potential was assayed from the livers of transplanted NOD-SCID mice and flow cytometry was used to show that the number of CD34^+^ and CD133^+^CD34^++^ liver cells correlated with the number of CFCs detected (Fig. 4C). Four of the 5 transplanted mice yielded colonies, whereas no colonies grew from an untransplanted mouse. The majority of the colonies were LPP-CFC derived, but HPP-CFC were detected in 4 mice (Fig. 4C and D).

### Hepatic engraftment by different sources of hematopoietic progenitors

**Figure 5:**
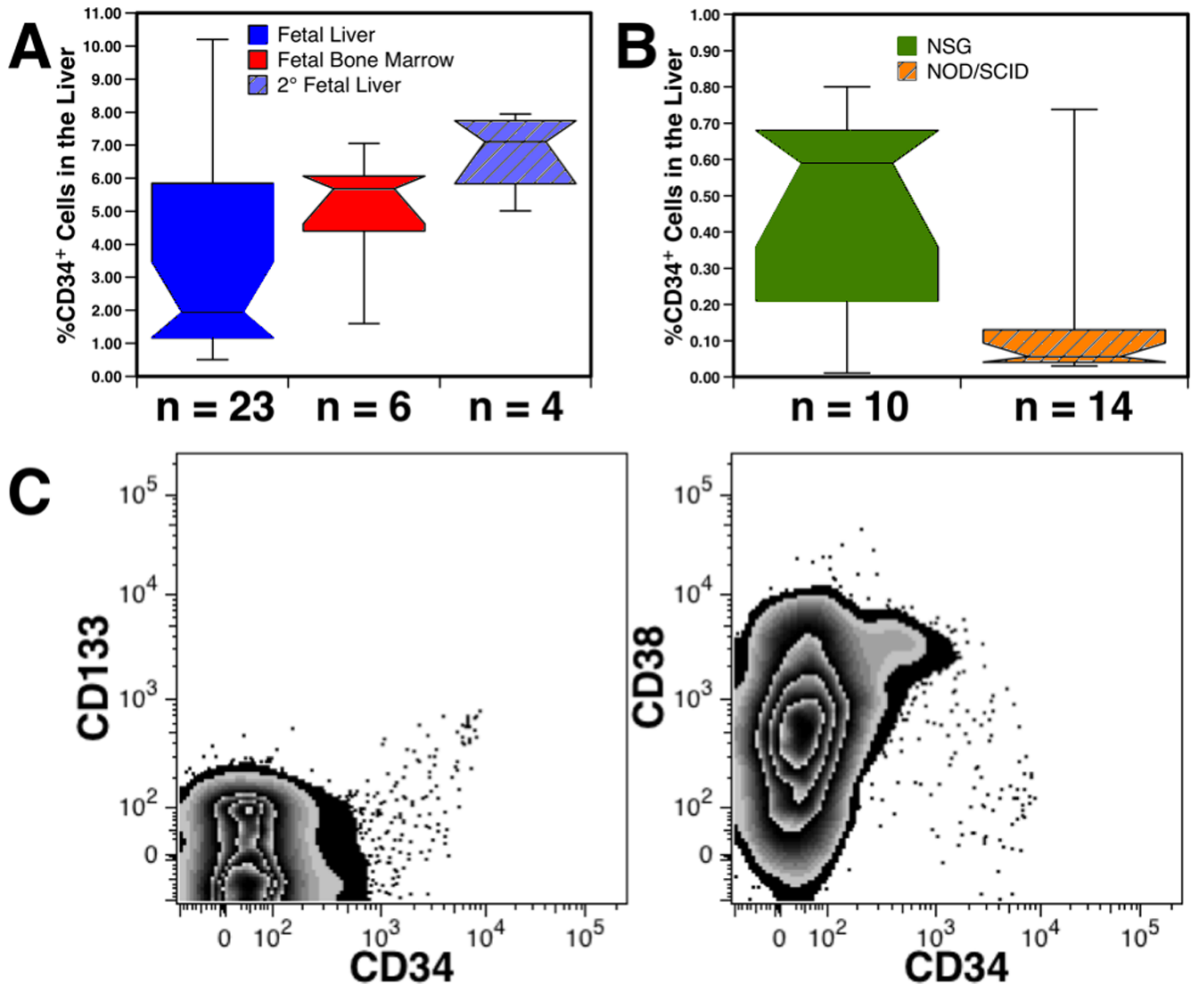
Liver engraftment by different sources of HSCs. (A) Engraftment of liver CD34^+^ cells after transplantation with 1×10^5^–2×10^6^ hFL or 2×10^7^ hFBM cells. Also shown is hepatic CD34^+^ cell reconstitution by secondary (2°) transplanted hFL cells obtained from the BM of the primary recipients. (B) Liver CD34^+^ cell engraftment is compared in NOD-SCID and NSG mice. Mice were analyzed 30 days after transplant with 1×10^7^ hFBM cells of 18 or 20 weeks' gestation. Data represent the frequency of CD34^+^ cells among all live cells. (C) An example of a low, but detectable, level of liver hematopoiesis observed 122 days after transplantation of 2×10^5^ UCB cells.

We evaluated NSG mice transplanted with different sources of HSCs to determine if the origin of the cells affected their ability to engraft the liver. Twenty-three mice were transplanted with 7 hFL preparations and analyzed 68–166 days after transplant (Fig. 5A). These were compared to mice transplanted with 2 different hFBM samples analyzed 85 and 92 days after transplant. There was no significant difference in the frequency of CD34^+^ cells found among light-density liver cells from mice transplanted with either hFL or hFBM, indicating that either source of HSCs could reconstitute liver hematopoiesis. Moreover, human hematopoiesis was observed in the livers of mice 31–32 weeks of age (166 days after transplant) demonstrating that the murine liver can support hematopoiesis well into adulthood.

Liver and BM engraftment was compared in chimeric mice to determine if there was any correlation between the frequencies of human cells found in the liver and the BM. Data were compiled from mice transplanted with either hFL or hFBM cells. The frequencies of human CD59^+^ cells in the BM of 29 transplanted mice varied over a broad range of 7.9 to 86.4%. The percentages of CD34^+^ cells among human cells in the BM of these samples ranged from 1.0–22.6%. Comparison of the BM data to the frequencies of CD34^+^ cells found in the liver (Fig. 5A) showed no correlation (data not shown). Cases of high chimerism in the liver were found associated with low levels of BM engraftment and vice versa. No case of liver engraftment was observed in the absence of any detectable BM engraftment.

Hematopoietic engraftment of the liver was also evaluated after secondary BM transplantation. Secondary recipients were transplanted with BM equivalent to the content of 36% of a mouse's femur harvested 68 days after the initial transplant with 1×10^6^ Lin^−^ LDFL cells. Human HSCs residing in the BM of the primary recipients had reconstituted liver hematopoiesis by 91 days after secondary transplant (Fig. 5A) to a similar extent as primary transplants of hFL and hFBM cells (P > 0.05).

Liver and BM engraftment were compared between NOD-SCID and NSG mouse strains (Fig. 5B). Median engraftment of human CD59^+^ cells in the BM of NOD-SCID mice was 0.62% (n  =  14) and 7.28% in NSG mice (n  =  10) (P < 0.001; data not shown). Similarly, the median percentage of CD34^+^ cells in the BM and liver was 4.4-fold (P  =  0.006) and 10.5-fold (P  =  0.008) higher, respectively, in NSG mice than in NOD-SCID mice (Fig. 5B). These results are consistent with past reports that the greater immunodeficiency of NSG mice, specifically their lack of NK-cells, makes this strain of mice a more permissive host for human cells than NOD-SCID mice [35].

To investigate if hematopoietic engraftment of the liver is a result of using fetal HSCs, we evaluated engraftment in mice transplanted with Lin^−^ UCB cells harvested at term (38–39 weeks' gestation). Four experiments were performed in which 2–6×10^5^ UCB cells were transplanted into 3 NSG mice for each experiment. Reconstitution was analyzed after 84–147 days. The median level of human engraftment (CD59^+^ cells) in the BM of the 12 mice was only 0.14% (range 0 - 5.5%). CD34^+^ cells were detected in the BM of only 8 of the 12 mice. Despite the low levels of BM engraftment, CD34^+^ cells were also detected in the livers of 5 mice and CD38^−^CD133^+^CD34^+^ cells were observed in 3 of these mice. Results from one of these mice are shown in (Fig. 5C). Although reconstitution with UCB cells was generally less vigorous than with fetal cells, liver engraftment was also observed using this source of neonatal HSCs.

### The liver contains transplantable HSCs

**Figure 6:**
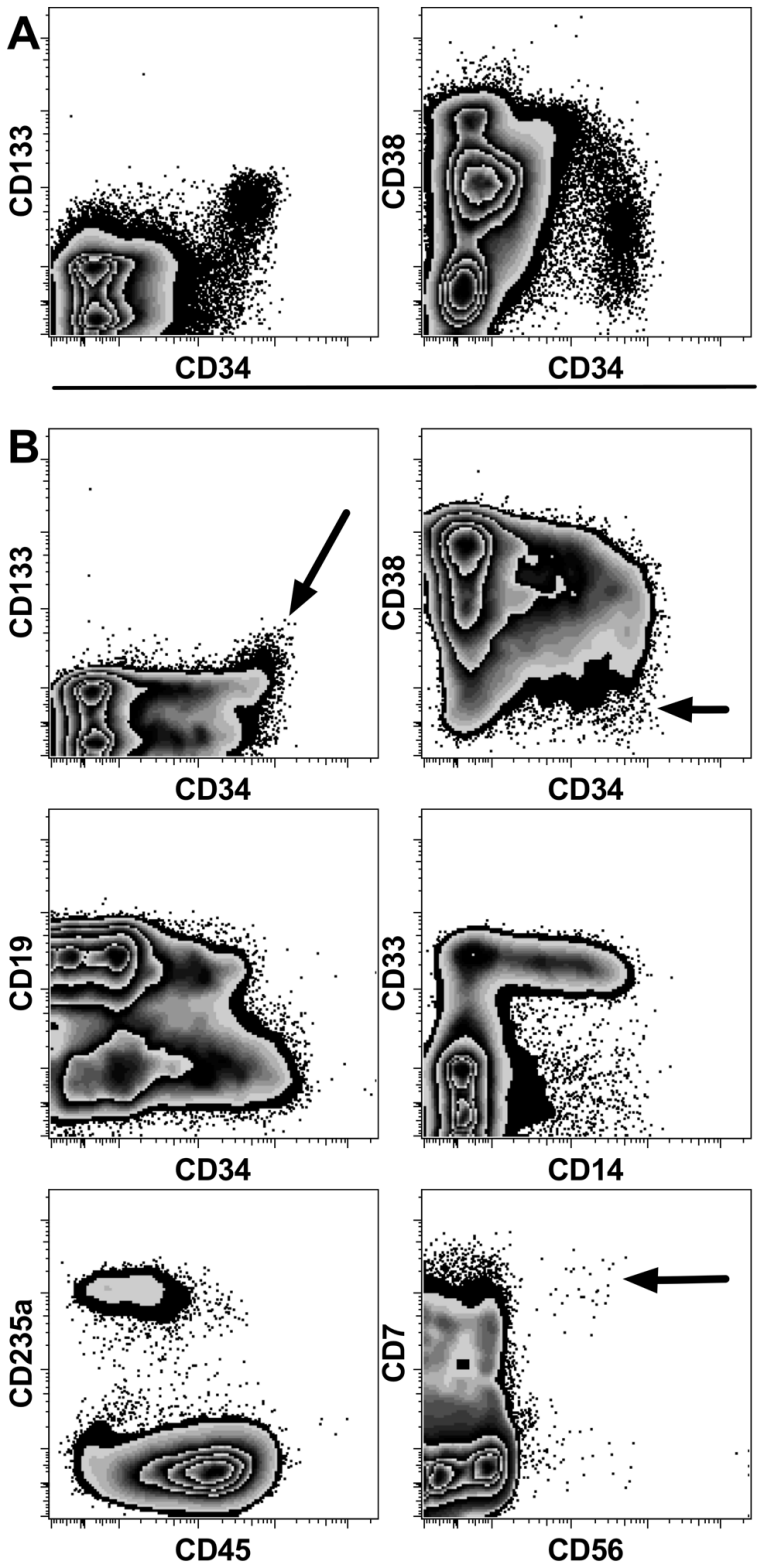
Hematopoietic reconstitution following transplantation of chimeric liver cells. (A) Phenotypic analysis of light density liver cells pooled from 9 mice harvested 89 days after transplant with hFBM cells reveals evidence of CD38^−^CD34^++^ and CD133^+^CD34^++^ cells. These cells were used for transplantation into secondary recipients. (B) An example of the multilineage reconstitution of the BM of a secondary recipient 69 days after transplantation with chimeric liver cells. Arrows identify CD38^−^CD34^++^ and CD133^+^CD34^++^ candidate HSCs and CD7^++^CD56^+^ NK cells.

Liver cells harvested from transplant recipients were re-transplanted to determine if HSCs capable of sustained multilineage hematopoietic reconstitution are present in the liver. Primary recipients were transplanted with 5×10^6^ total hFBM cells (22 weeks' gestation) and the liver cells were harvested 89 days post transplant. Analysis of liver cells pooled from 9 mice indicated that CD38^−^CD34^++^ and CD133^+^CD34^++^ cells were in the cell preparation, suggesting the presence of HSCs (Fig. 6A). Secondary hosts were transplanted with the equivalent of 90% of a single liver's content of light-density cells and engraftment was analyzed 69 days later (Fig. 6B). Full hematopoietic reconstitution was observed in 2 of 3 transplanted mice. Human BM chimerism rates in the 3 mice were 0.1%, 3.0% and 7.9%. The recipient with the lowest level of chimerism lacked detectable erythroid reconstitution but myeloid, B-cell and CD34^+^ cell engraftment was detected. Moreover, in the recipient with the highest level of human cells, CD38^−^CD34^++^ and CD133^+^CD34^++^ HSCs and CD7^++^CD56^+^ NK-cells were observed (Fig. 6B). These findings indicate that transplantable HSCs were among the donor liver cells.

### Hematopoiesis in the livers of old mice

Since our findings with the xenogeneic transplant model point to the murine liver retaining hematopoietic function throughout life, we examined if murine hematopoiesis could be detected in very old mice. Hepatic hematopoiesis has not been previously examined in very old mice and our findings with immunodeficient mice may be affected by the perturbed hematopoiesis in these mice as well as species-specific factors affecting human hematopoiesis in the murine liver.

**Figure 7:**
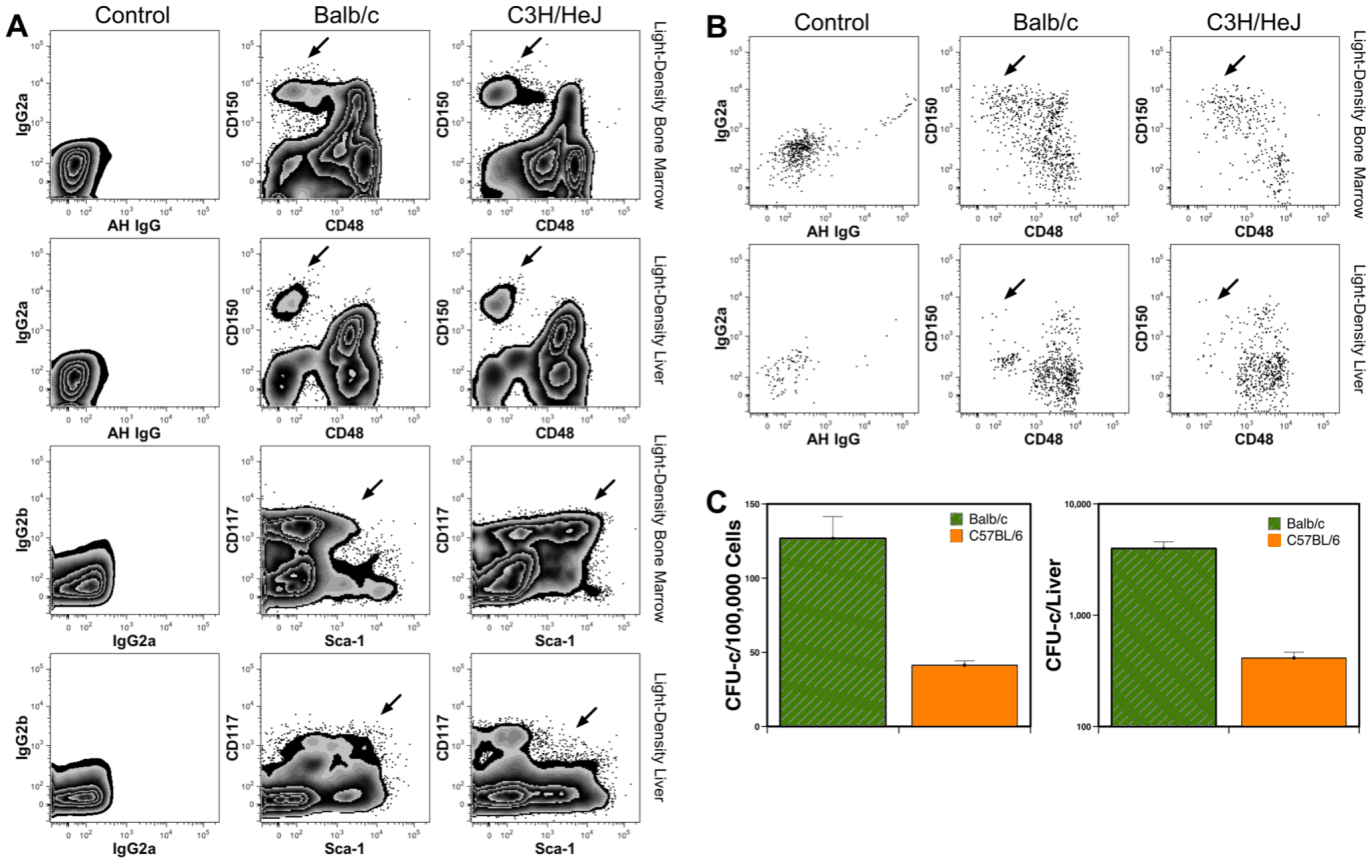
Hematopoietic stem and progenitor cells are present in the livers of old mice. Phenotypic analyses of hematopoietic stem cells and progenitors present in the light-density fraction of BM and livers of 2-year old Balb/cJ or 1-year old C3H/HeJ mice (A). Arrows identify populations of either CD48^−^CD150^+^ or Sca-1^+^CD117^+^ cells. Data depicted include live, single cells based on lack of PI staining and low lineage expression. Data from Balb/cJ and C3H/HeJ strains are compared to a negative isotype control shown in the left column. The Sca-1^+^CD117^+^ population contains a CD48^−^CD150^+^ population in both the BM and liver of old mice (B). Events were gated for Sca-1^+^CD117^+^Lin^−^ single-live cells. Myeloid CFU-c were measured among light-density cells harvested from livers of 1-year old Balb/c and C57BL/6 mice (C). CFU-c per liver was calculated based on the frequencies of CFU-c shown and cell counts. Results are shown as the mean measurements on 3 or 4 mice of each strain. Note that the graph on the right represents data shown on a logarithmic scale.

Liver cells were harvested from 2-year old Balb/cJ and 1-year old C3H/HeJ mice. Flow cytometric analysis revealed expression of CD48 and CD150 among lineage-depleted liver cells that shared similarity with the pattern observed in the BM (Fig. 7A). Murine HSCs are found among CD48^−^CD150^+^ cells [36], which were observed in the livers of both strains of old mice. Low number of Sca-1^+^CD117^+^ lineage-depleted cells, likely representing hematopoietic precursors [37], were also detected among liver cells. Gating on these Sca-1^+^CD117^+^ cells further revealed a small population of CD48^−^CD150^+^ cells in the liver, thought to be highly enriched in HSCs (Fig. 7B) [36]. In addition, myeloid CFU-c were detected from year-old Balb/cJ and C57BL/6J mice, with a trend towards higher numbers being detected in Balb/cJ mice. Observation of CFU-c supports the phenotypic evidence indicating ongoing hematopoiesis in the livers of old mice.

### Hematopoietic engraftment by intra-splenic injection without cytoablation in uPA-NOG mice

We evaluated the potential to further humanize our murine model of hepatic hematopoiesis by performing hFL transplants on uPA-NOG mice. The urokinase-type plasminogen activator (uPA)-transgene expressed in the liver of these mice, under an albumin promotor, has been shown to confer an engraftment advantage for adult human hepatocytes, resulting in the formation of a chimeric liver [26]. Our aim was to determine if engraftment of non-hematopoietic elements, such as hepatocytes, could be achieved to allow for study of the interactions between these cells and the human hematopoietic cells in a small animal model.

**Figure 8:**
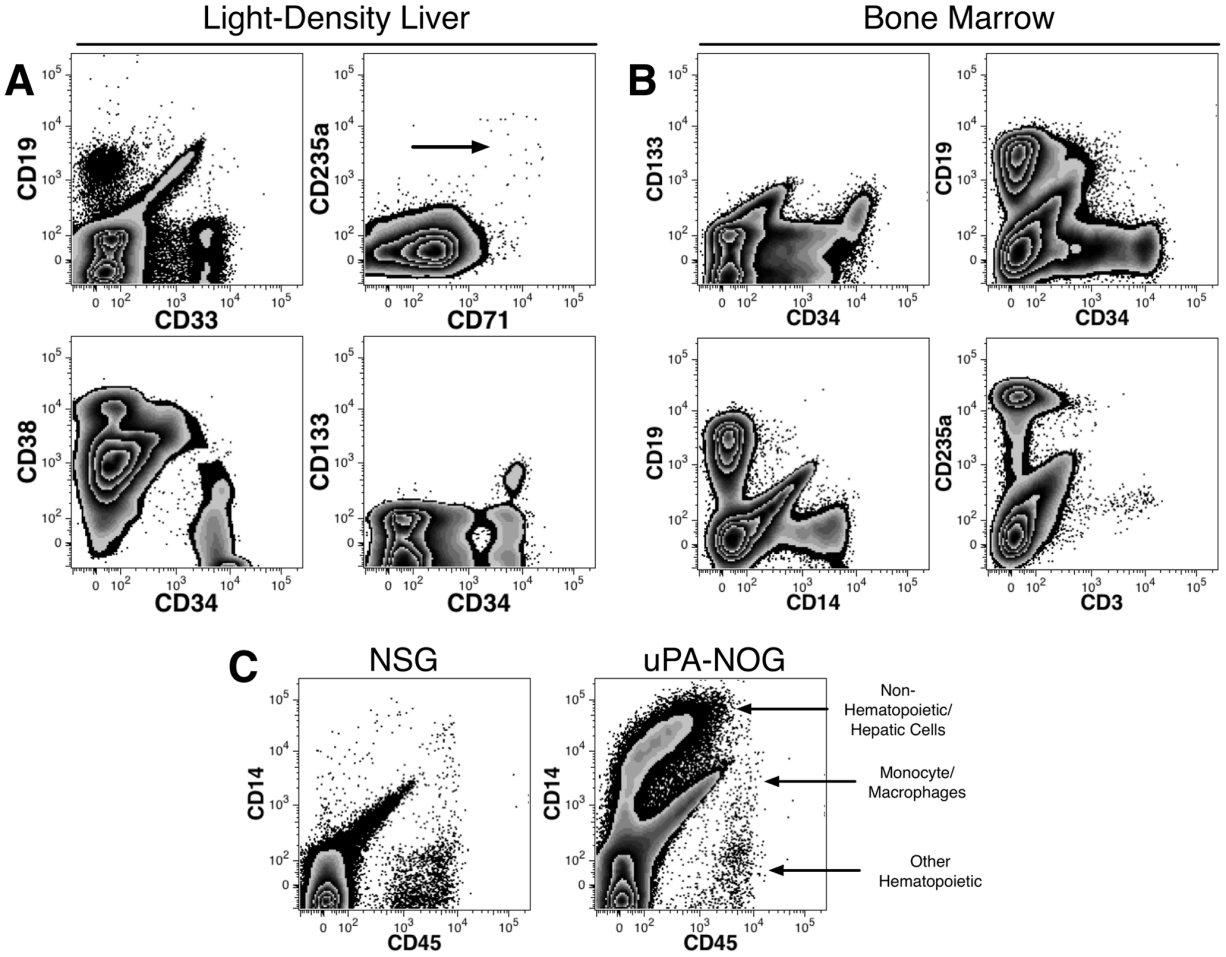
Hematopoietic reconstitution of uPA-NOG mice. (A) Adult mice were transplanted with erythrocyte-depleted hFL cells by intra-splenic injection. No irradiation was used for pre-transplant cytoablation. Engraftment was evaluated 75 - 82 days after transplant. Digested liver cell suspensions were separated into quickly-settling high-density and the remaining, low-density, cells. The light-density cells were analyzed for hematopoietic reconstitution. Note the presence of CD19^+^ B-cells, CD33^+^ myeloid cells, possible immature erythroid elements (arrow, CD71^++^CD235a^+^ cells) and CD34^+^ hematopoietic stem (CD38^−^CD133^+^) and progenitor cells (CD38^+^CD133^−^). (B) Multilineage hematopoietic engraftment was also observed in the BM of a uPA-NOG mouse. (C) High-density cells isolated from uPA-NOG transplanted mice contained CD45^−^CD14^+^ cells likely representing liver endothelial cells as well as CD45^+^ hematopoietic cells (C). The same population of CD45^−^CD14^+^ cells was much less prevalent in NSG mice.

Engraftment of uPA-NOG mice was achieved by intra-splenic transplants without the use of irradiation for cytoablation owing to the delicate health status of this strain of mice. Liver engraftment was evaluated 75 to 82 days after transplant of erythrocyte-depleted LDFL cells in 4 uPA-NOG mice. The light-density cells, collected by bench-top sedimentation, were analyzed for hematopoietic reconstitution. Abundant CD19^+^ B-cells, CD33^+^ myeloid cells and a small population of CD71^++^CD235a^+^ erythroid precursors were observed (Fig. 8A). Potential HSCs (CD38^−^CD34^++^ and CD133^+^CD34^++^) and committed progenitors (CD38^+^CD34^+^ and CD133^−^CD34^+^) were also detected.

Hematopoietic engraftment following intra-splenic transplantation was also evaluated in the BM and spleens of uPA-NOG and NSG mice. In the BM, human CD59^+^ cells represented a range of 0.7–5.6% in uPA-NOG (n  =  4) and 0.4–25% in NSG (n =  3) mice. Multilineage engraftment was detected in 3 of the 4 uPA-NOG mice and 2 of the 3 NSG mice as evinced by the detection of CD235a^+^ erythrocytes, CD14^+^ monocytes, CD19^+^ B-cells and CD34^+^ progenitors (Fig. 8B). Erythrocytes were undetectable in the fourth uPA-NOG mouse, despite the presence of the other cell types. B-cells were the only cell type positively identified in the third NSG mouse with partial engraftment. Thus, hematopoietic engraftment can be achieved in immunodeficient mice using intra-splenic transplantation without prior cytoablation.

### Liver engraftment of uPA-NOG mice by non-hematopoietic cells

Quickly sedimenting, high-density cells from uPA-NOG mice were found to contain a population of CD45^−^CD14^+^ cells that was less prevalent in NSG mice (Fig. 8C). CD45^+^ cells, including a few CD45^+^CD14^+^ monocytic cells, were observed in both strains of mice. Although CD14 is expressed on hepatocytes, recent evaluation of these CD45^−^ cells indicated that they were liver sinusoidal endothelial cells (LSECs), not hepatocytes [27].

**Figure 9:**
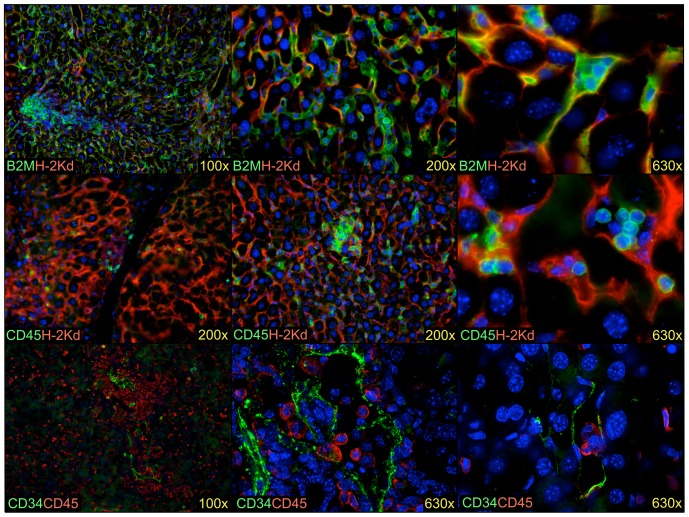
Human cells engrafted in the mouse liver. Human B2M^+^ cells (green) are seen growing as colonies in the parenchyma (top row). These cells are small elongated cells located around mouse hepatocytes and lining sinusoids, indicative of LSECs. Human CD45^+^ leukocytes (green) are found dispersed throughout the liver parenchyma as well as in some blood vessels (middle row). Note the presence of small clusters of leukocytes. LSECs stain brightly for CD34 (green) and are shown to be in close contact with the human CD45^+^ leukocytes (bottom row). Mouse cells are stained in the top two rows using anti-H-2K^d^ (red) and blue staining represents nuclei stained with DAPI. The fold-magnification used is indicated for each photograph.

CD45^+^ cells, including a few CD45^+^CD14^+^ monocytic cells, were also observed in both strains of mice. Staining of liver sections shows CD45^+^ cells dispersed throughout the liver, sometimes in small clusters, as well as in the vessels and the liver sinusoids (Fig. 9). Additionally, LSECs growing in colonies surrounding mouse hepatocytes were observed corresponding to the CD45^−^CD14^+^ cells observed by flow cytometry. High magnification photomicrographs reveal association of small round leukocyte populations with both murine and human non-hematopoietic cells. Moreover, the human LSECs, identified by CD34 expression, were observed to directly interact with CD45^+^ cells in the sinusoids of the mouse liver.

## Discussion

The liver plays a vital role in hematopoiesis during prenatal development and has been known to contribute to extramedullary hematopoiesis during times of hematopoietic insufficiency. However, little is known of the regulation of hepatic hematopoiesis in adults and its contribution to steady-state and stressed hematopoiesis. It is believed that HSCs reside in the adult liver. In mice, it has been shown that young adults harbor transplantable stem cells in the liver [11]–[13]. We extend this finding to show that even mice as old as 2 years have evidence of ongoing hepatic hematopoiesis. In humans, the data supporting the presence of HSCs in the liver rests on the observation of cells with the phenotypic characteristics of HSCs [15] and the observations of hematopoietic chimerism that can arise, in some cases, after liver transplantation [18]–[20]. Herein, we demonstrate that the adult murine liver can support a population of human HSCs and their development into multiple blood cell lineages. The existence of HSCs in the liver is supported by observations made by flow cytometric phenotyping, assay of HPP-CFC and, most convincingly, by the functional observation that liver cells can reconstitute BM hematopoiesis in secondary transplant recipients.

The evidence for ongoing human hematopoiesis in the murine livers includes the observation of a spectrum of CD34 expression as well as expression of CD38, a marker of committed hematopoietic progenitors [6], [38]. High levels of B-lymphopoiesis were indicated by the co-expression of CD34 and CD19 [39]. Murine livers also supported a population of mature CD14^+^ monocytic cells, which may have developed from CD33^+^CD34^+^ myeloid progenitors found in the livers [40]. However, very few CD15^+^ cells were observed, indicating a general lack of granulopoiesis in the liver [41]. In this regard, murine Gr-1^+^Mac-1^+^ cells have been observed in small numbers in the mouse liver parenchyma possibly representing mature granulocytes [42]. Alternatively, these cells could instead represent immature myeloid cells [43] and may, therefore, be the precursors of the comparatively more abundant monocytic cells found in the liver. We detected human LPP-CFC with myeloid lineage-potential as predicted by the flow cytometric analyses. CD14 expression occurs late in the development of monocytes, after the loss of CD34 expression, so does not mark a progenitor population [44]. Nonetheless, the spectrum of CD14 expression observed is further evidence of ongoing monocyte maturation occurring in the liver.

Mature T-cells and NK-cells were present in some livers, but these cells may have developed in other hematopoietic tissues or survived from the initial graft and subsequently migrated to the liver [42]. Nonetheless, the observation of DP CD4^+^CD8^+^ T-cells in the liver, like those found in the thymus, supports previous observations that the murine liver can be a site of human T-lymphopoiesis [45]. Studies in mice have also shown the liver to be a site of extrathymic T-lymphopoiesis [12]. The murine hepatic T-cells tend to express CD122, intermediate levels of the T-cell receptor and about half belong to the subset of NKT-cells expressing the NK1.1 antigen. Other less common subsets of T-cells found enriched in the murine liver include γδ T-cells and DN T-cells (CD3^+^CD4^−^CD8^−^ cells) [46], [47]. We did not evaluate T-cell receptor expression in our humanized mice, but our analysis of CD4 and CD8 expression did not reveal a sizable subset of DN T-cells. Indeed, most T-cells were of the DP phenotype as also observed by Choi et al. after intrahepatic xenotransplantation [45].

Human mast cells were detected in the livers of transplanted mice as in the human liver where they are believed to play diverse roles in liver disease and transplant rejection [31]. Mast cells are specifically identified by their expression of CD203c and high levels of CD117, the receptor for KL [34]. Indeed, KL is a critical factor for the development of mast cells and mice deficient in this cytokine lack mast cells [48]. Murine KL can support the growth of human cells [49], and this growth factor is known to be expressed in the liver, where it also plays a role in the growth of hepatocytes [50], [51]. The hepatic mast cells may have, therefore, developed from engrafted hematopoietic precursors either in the BM or directly in the liver. This supported by our observation that CD38^−^CD34^++^ cells gave rise to mast cells. Additionally, some mature mast cells in the graft may have survived and proliferated in the murine liver. The hFL is a known source of mast cell precursors from which mast cells can be readily grown in culture [52]. To our knowledge, however, mature mast cells have not been previously reported in the midgestation fetus. Analysis of hFBM and hFL cells revealed a population of cells with the phenotypic characteristics like those of mast cells cultured from hFL [32], [33]: these cells expressed the common leukocyte antigen CD45, myeloid antigens CD13 and CD33, CD9, the adhesion molecules CD49d and CD49e, and only very low levels of the IgE receptor. Our findings demonstrate that hepatic and BM mast cells develop prenatally in human ontogeny and their survival and possible development, is supported by the murine liver.

Our observation that the adult mouse liver supports human hematopoiesis and is populated by a spectrum of mature human blood cells offers the possibility to study human hepatic hematopoiesis and hepatic immune functions in an animal model. However, some caution in interpretation of findings in this xenogeneic mouse model are warranted. A number of murine hematopoietic growth factors such as IL-3, IL-4, IL-15, M-CSF and GM-CSF are not active on human cells and, thus, the multiple stages of hematopoiesis are likely to be affected in particular the myeloid, erythroid and NK-cell lineages [53]. For instance, human monocytes found in the liver must be supported by growth factors other than murine M-CSF and GM-CSF. Engrafted hFBM cells were shown to produce cytokines, including M-CSF, raising the possibility that hepatic monocyte development is supported by human growth factors [54]. Robust human B-lymphopoiesis was also observed, but whether this was a consequence of the underlying immunodeficiency of the host is not clear. Serum levels of murine IL-7, a critical B-cell growth factor, are undetectable in NSG mice [55], but elevated localized production of this cytokine in the liver is a possibility.

During fetal development, the human liver is exceptionally erythropoietic [3], [4], which wanes early in life. We observed only low levels of erythropoiesis in the adult chimeric liver. However, previous reports have noted depressed human erythropoiesis in chimeric mice [56], and our own experience analyzing the BM of chimeric NSG mice confirms a trend toward lower levels of erythropoiesis than either myelopoiesis or B-lymphopoiesis (Fig. 7 and unpublished data). There is also controversy as to whether murine erythropoietin is as active on human as mouse cells [57]-[59]. Any possible deficiency in EPO stimulation may also be compounded by the lack of cross-reactivity of murine GM-CSF and other growth regulatory molecules leading to depressed human erythropoiesis in the murine BM and liver. Moreover, oncostatin M is thought to play a major role in the inhibition of post-natal erythropoiesis and hepatic hematopoiesis in general [60].

Mice with humanized livers play an important role in the study of liver disease [61]. Animal models for human diseases are needed to reproduce and study the complexity of cellular interactions that take place through all the stages of pathology and its resulting immune response. As the liver is comprised of many different cellular elements, the challenge is to construct humanized mice that have both liver parenchymal cells as well as a fully functional immune system. We recently described the uPA-NOG mouse as a permissive host for adult, but not fetal, human hepatocytes [26], [27] and, herein, we demonstrate hematopoietic reconstitution of these mice as well. When transplanted with hFL cells, LSECs can engraft at much higher levels in uPA-NOG mice than NSG mice. We show interaction of these LSECs with leukocytes in the liver sinusoids, which should allow future study of the in vivo interactions of these cells and the potential role of LSECs in hematopoiesis. We are also currently pursuing methods to foster fetal hepatocyte engraftment in uPA-NOG mice to bring us a step closer to being able to construct mice with livers containing all the cellular elements found in the human liver.

## Supporting Information

Figure S1 **Human engraftment in the livers of mice with indication of graft versus host disease.** Light-density liver cells were pooled from 10 livers harvested 321 days after transplantation of NSG mice with 2×10^7^ FBM cells. Mice exhibited extensive hair loss and/or enlarged spleens suggestive of graft versus host disease. Numbers shown in the plots indicate the percentage of parental events in the indicated gate. (A) The pooled liver cells were analyzed for the presence of live-single human cells as indicated. (B) Multiple hematopoietic lineages were detected among the human cells, including a high frequency of T-cells. All numbers shown in the plots indicate the percentage of gated events observed among live-single human cells.
(PDF)

Table S1 **Antibodies used in this study.**
(PDF)

Checklist S1 **The ARRIVE Guidelines Checklist.**
(PDF)
