# Supplementary material for: The Adult Livers of Immunodeficient Mice Support Human Hematopoiesis: Evidence for a Hepatic Mast Cell Population that Develops Early in Human Ontogeny
Source: PLoS One. 2014 May 12;9(5):e97312. doi: 10.1371/journal.pone.0097312 (PMC4018295; doi:10.1371/journal.pone.0097312)
Supplement: Figure S1 — Human engraftment in the livers of mice with indication of graft versus host disease. Light-density liver cells were pooled from 10 livers harvested 321 days after transplantation of NSG mice with 2×10^7^ FBM cells. Mice exhibited extensive hair loss and/or enlarged spleens suggestive of graft versus host disease. Numbers shown in the plots indicate the percentage of parental events in the indicated gate. (A) The pooled liver cells were analyzed for the presence of live-single human cells as indicated. (B) Multiple hematopoietic lineages were detected among the human cells, including a high frequency of T-cells. All numbers shown in the plots indicate the percentage of gated events observed among live-single human cells. (PDF) [file pone.0097312.s001.pdf]

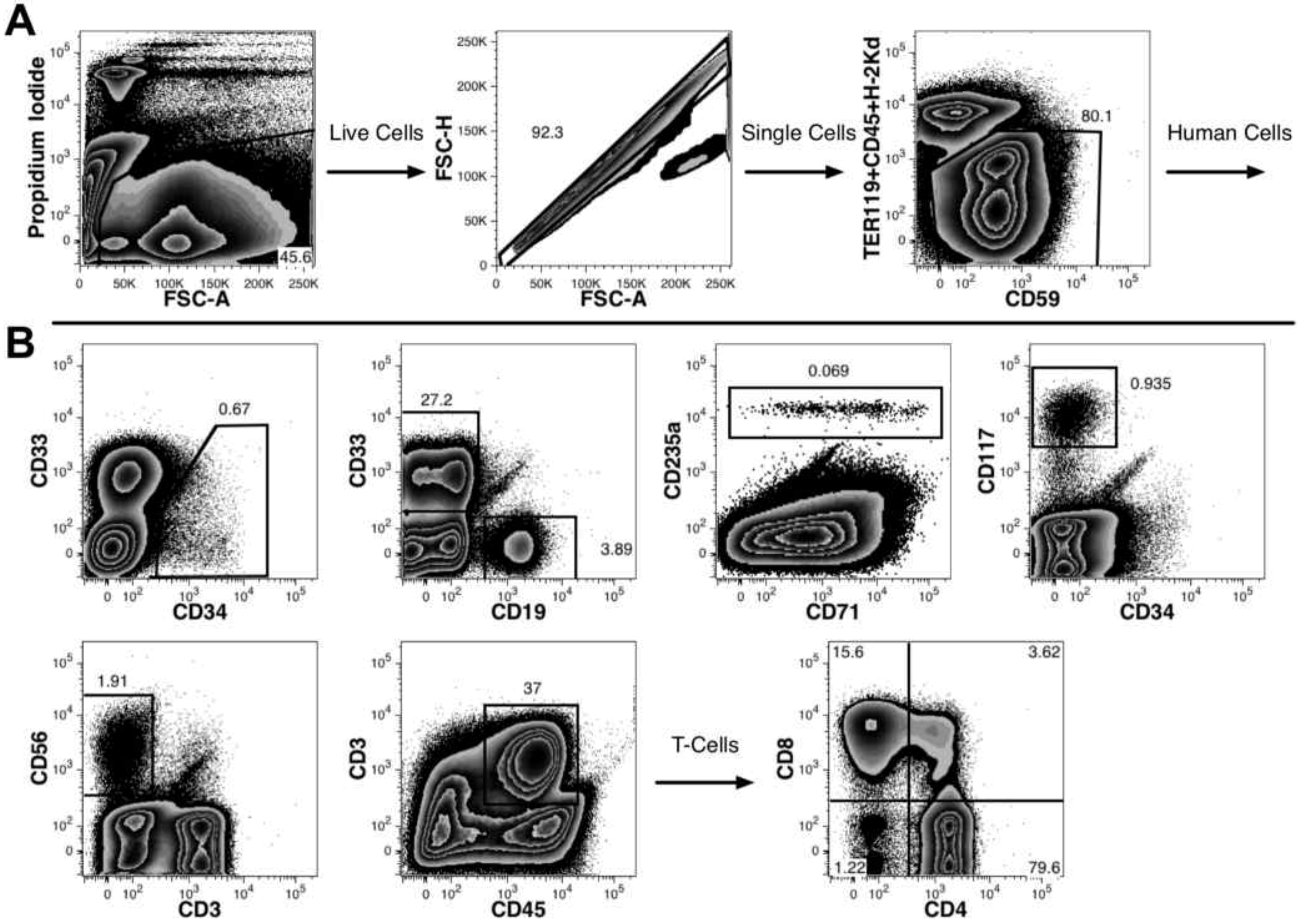

Figure S1. Human engraftment in the livers of mice with indication of graft versus host disease. Light-density liver cells were pooled from 10 livers harvested 321 days after transplantation of NSG mice with 2 x 10^7 FBM cells. Mice exhibited extensive hair loss and/or enlarged spleens suggestive of graft versus host disease. Numbers shown in the plots indicate the percentage of parental events in the indicated gate. (A) The pooled liver cells were analyzed for the presence of live-single human cells as indicated. (B) Multiple hematopoietic lineages were detected among the human cells, including a high frequency of T-cells. All numbers shown in the plots indicate the percentage of gated events observed among live-single human cells.
