# Supplementary material for: The Adult Livers of Immunodeficient Mice Support Human Hematopoiesis: Evidence for a Hepatic Mast Cell Population that Develops Early in Human Ontogeny
Source: PLoS One. 2014 May 12;9(5):e97312. doi: 10.1371/journal.pone.0097312 (PMC4018295; doi:10.1371/journal.pone.0097312)
Supplement: Table S1 — Antibodies used in this study. (PDF) [file pone.0097312.s002.pdf]

Table S1. Antibodies used in this study.

Specificity-Label	Clone	Product #	Isotype	Source
		Antibodies recognizing human antigens		
CD3	SK7	347340	IgG1	BD Biosciences, San Jose, CA
CD3-FITC	UCHT1	300406	IgG1	BioLegend, San Diego, CA
CD3-PE/Cy7	UCHT1	300420	IgG1	BioLegend
CD4-PE	IV T114	550630	IgG1	BD Biosciences
CD4-APC	RPA-T4	300514	IgG1	BioLegend
CD7-PE	CD7-6B7	MHCD0704	IgG2a	Life Technology, Carlsbad, CA
CD7-PE	CD7-6B7	343106	IgG2a	BioLegend
CD8-PE	RPA-TA	557086	IgG1	BD Biosciences
CD8-APC	SK1	344722	IgG1	BioLegend
CD8-FITC	SK1	344704	IgG1	BioLegend
CD9-PE	M-L13	555372	IgG1	BD Biosciences
CD13-PE	L138	347837	IgG1	BD Biosciences
CD13-APC	WM15	301706	IgG1	BioLegend
CD13-FITC	WM15	301704	IgG1	BioLegend
CD14	RPA-M1		IgG1	Produced from cell culture.
CD14-FITC	HCD14	325604	IgG1	BioLegend
CD14-FITC	Tük4	MHCD1401	IgG2a	Life Technology
CD14-APC	HCD14	325-608	IgG1	BioLegend
CD14-PE/Cy7	HCD14	325618	IgG1	BioLegend
CD15-FITC	W6D3	323004	IgG1	BioLegend
CD16-PE	3G8	IM1238	IgG1	Beckman Coulter, Fullerton, CA
CD19	4G7	347540	IgG1	BD Biosciences
CD19-FITC	SJ25-C1	MHCD1901	IgG1	Life Technology
CD19-PE	HIB19	302208	IgG1	BioLegend
CD19-PC7	HIB19	302216	IgG1	BioLegend
CD20	L27	347670	IgG1	BD Biosciences
CD20-FITC	HI47	MHCD2001	IgG3	Life Technology
CD33-PE	P67.6	347787	IgG1	BD Biosciences
CD33-APC	WM53	303408	IgG1	BioLegend
CD34-FITC	581	555821	IgG1	BD Biosciences
CD34-PE	581	CD34-581	IgG1	Life Technology
CD34-APC	581	343510	IgG1	BioLegend
CD34-PE/Cy7	581	343516	IgG1	BioLegend
CD34	561	34602	IgG2a	BioLegend
CD38-PE	HB7	347687	IgG1	BD Biosciences
CD45-FITC	H130	304006	IgG1	BioLegend
CD45-PE	H130	304008	IgG1	BioLegend
CD45-PE/Cy7	H130	304016	IgG1	BioLegend
CD45-APC-H7	2D1	560178	IgG1	BD Biosciences
CD49d-PE	9F10	304304	IgG1	BioLegend
CD49e-PE	NKI-SAM-1	328010	IgG2b	BioLegend
CD56	MY31	347740	IgG1	BD Biosciences
CD56-FITC	C5.9	562	IgG2b	Exalpha Biologicals, Inc., Boston, MA
CD59-FITC	p282	304706	IgG2a	BioLegend
CD56-APC	HCD56	318310	IgG1	BioLegend
CD59-AF700	p282 (H19)	Custom Product	IgG2a	BioLegend
CD71-PE	L01.1	555537	IgG2a	BD Biosciences
CD71-PE	YDJ1.2.2	IM2001	IgG1	Beckman Coulter
CD105-PE	SN6	MHCD10504	IgG1	Life Technology
CD105-APC	43A3	323208	IgG1	BioLegend
CD117-APC	104D2	313206	IgG1	BioLegend
CD117-APC	104D2	341096	IgG1	BD Biosciences
CD133-APC	AC133	130-090-826	IgG1	Miltenyi Biotec, Auburn, CA
CD133-PE	AC133	130-080-801	IgG1	Miltenyi Biotec
CD161-APC	HP-3G10	339912	IgG1	BioLegend
CD161-PE	HP-3G10	339904	IgG1	BioLegend
CD203c-PE	NP4D6	324606	IgG1	BioLegend
CD203c-APC	FR3-16A11	130-092-344	IgG1	Miltenyi Biotec
CD235a	10F7MN		IgG1	Produced from cell culture.
CD235a-FITC	CLB-ery-1	MHGLA01	IgG1	Life Technology
CD235a-PE	CLB-ery-1	MHGLA04	IgG1	Life Technology
FcεRIα-PE	AER-37 (CRA1)	12-5899	IgG2b	eBioscience, San Diego, CA
HLA-DR-PE	TÜ36	555561	IgG2b	BD Biosciences
		Non-specific antibodies for isotype control		
Mouse IgG1-APC	MOPC-21	400120	IgG1	BioLegend
Mouse IgG1-FITC	MOPC-21	400108	IgG1	BioLegend
Mouse IgG1-PE	MOPC-21	400114	IgG1	BioLegend
Mouse IgG1-PE/Cy7	MOPC-21	400126	IgG1	BioLegend
Mouse IgG2a-APC	MOPC-173	400220	IgG2a	BioLegend
Mouse IgG2a-FITC	MOPC-173	400208	IgG2a	BioLegend
Mouse IgG2a-PE	G155-178	559319	IgG2a	BD Biosciences
Mouse IgG2b-PE	27-35	555743	IgG2b	BD Biosciences
Mouse IgM-APC	MM-30	401616	IgM	BioLegend
		Antibodies recognizing mouse antigens		
CD45	30-F11	103126	IgG2b	BioLegend
$H2\text{-}K^d$	SF1-1.1	116616	IgG2a	BioLegend
TER-119	TER-119	116232	IgG2b	BioLegend
